# Microstructure, Mechanical Properties, and Tribological Behavior of Friction Stir Lap-Welded Joints Between SiC_p_/Al–Fe–V–Si Composites and an Al–Si Alloy

**DOI:** 10.3390/ma18153589

**Published:** 2025-07-30

**Authors:** Shunfa Xiao, Pinming Feng, Xiangping Li, Yishan Sun, Haiyang Liu, Jie Teng, Fulin Jiang

**Affiliations:** 1 College of Materials Science and Engineering, Hunan University, Changsha 410082, China; xiaoshunfa2024@163.com (S.X.); pmfeng@hnu.edu.cn (P.F.); tengjie@hun.edu.cn (J.T.); 2 National Key Laboratory of Science and Technology on High-Strength Structural Materials, Central South University, Changsha 410083, China; 3 CRRC Qishuyan Institute Co., Ltd., Changzhou 231011, China; lixiangping0407@163.com (X.L.); sun1sandon@163.com (Y.S.); 4 Hunan Province Engineering Research Center for the Preparation and Application of High Performance Aluminum Matrix Composites, Hunan Everrich Composite Corp, Luxi 416100, China

**Keywords:** friction stir lap welding, aluminum matrix composites, mechanical properties, tribological behavior

## Abstract

Aluminum matrix composites provide an ideal solution for lightweight brake disks, but conventional casting processes are prone to crack initiation due to inhomogeneous reinforcement dispersion, gas porosity, and inadequate toughness. To break the conventional trade-off between high wear resistance and low toughness of brake disks, this study fabricated a bimetallic structure of SiC_p_/Al–Fe–V–Si aluminum matrix composite and cast ZL101 alloy using friction stir lap welding (FSLW). Then, the microstructural evolution, mechanical properties, and tribological behavior of the FSLW joints were studied by XRD, SEM, TEM, tensile testing, and tribological tests. The results showed that the FSLW process homogenized the distribution of SiC particle reinforcements in the SiC_p_/Al–Fe–V–Si composites. The Al_12_(Fe,V)_3_Si heat-resistant phase was not decomposed or coarsened, and the mechanical properties were maintained. The FSLW process refined the grains of the ZL101 aluminum alloy through recrystallization and fragmented eutectic silicon, improving elongation to 22%. A metallurgical bond formed at the joint interface. Tensile fracture occurred within the ZL101 matrix, demonstrating that the interfacial bond strength exceeded the alloy’s load-bearing capacity. In addition, the composites exhibited significantly enhanced wear resistance after FSLW, with their wear rate reduced by approximately 40% compared to the as-received materials, which was attributed to the homogenized SiC particle distribution and the activation of an oxidative wear mechanism.

## 1. Introduction

Aluminum matrix composites (AMCs) are regarded as ideal substitutes for brake disk materials due to their high strength, excellent thermal conductivity, and superior corrosion resistance. In addition, aluminum matrix composites exhibit versatile applications across automotive and aerospace industries. Within the automotive sector, they have been successfully implemented in manufacturing components such as clutches, brakes, and mechanical seals. Moreover, critical parts including pistons, cylinder blocks, brake disks, and connecting rods demonstrate significant potential for further development [1]. Current research on the fabrication of composite brake disks predominantly focuses on stir-casting monolithic forming techniques. However, due to the inherent limitations of ceramic reinforcements, specifically their high surface tension and poor interfacial wettability, this process is prone to inducing typical defects such as gas entrapment, shrinkage porosity and segregation, and particle agglomeration. These defects consequently lead to the deterioration of mechanical properties. Furthermore, aluminum matrix composites suffer from insufficient toughness. During braking applications, the stress concentration effect induced by reinforcement fracture triggers microcrack nucleation and propagation, ultimately resulting in a decline in the contact fatigue life of the brake disk. Regarding the issue of the strength–toughness inversion relationship, scholars both domestically and internationally have dedicated considerable efforts [2,3,4] aimed at overcoming this inherent conflict. Among these approaches, heterogeneous lamination and cross-scale structural design strategies represent one significant breakthrough direction. As demonstrated by Li et al. [5], gradient sintering was employed to fabricate WC–Co alloys featuring a hard outer layer and a tough inner layer, leveraging the distinct advantages of each component. Furthermore, Du et al. [6], fabricated a SiC_p_/AZ91 magnesium-based laminated composite, achieving a compressive strength of 622 MPa. This represents a remarkable enhancement of 6000% and 200%, respectively, when compared to the monolithic SiC lattice structure (10.2 MPa) and the AZ91 alloy matrix (316 MPa). The high-density SiC_p_/AZ91 layers significantly enhanced the strength of the composite laminate. Concurrently, the AZ91 alloy layers not only mitigated stress concentration but also suppressed crack initiation and propagation, thereby imparting enhanced toughness to the composite.

Welding of dissimilar aluminum alloys has consequently garnered significant attention due to its potential to maximize the advantages inherent in different aluminum alloy grades. However, conventional welding techniques, such as brazing and tungsten inert gas (TIG) welding, tend to introduce defects like porosity and cracks into the joint. This consequently leads to the deterioration in joint performance [7,8]. Friction stir welding (FSW) is a novel solid-state joining technique characterized by high efficiency, environmental friendliness, elimination of shielding gas requirements, and low cost. Crucially, while avoiding the defect issues associated with conventional fusion welding processes, FSW has gained widespread application [9,10]. However, to date, research reports on the friction stir welding of dissimilar materials involving aluminum matrix composites and other materials—particularly aluminum alloys—remain limited. The primary research challenge currently lies in the substantial differences in physical properties (e.g., thermal conductivity, coefficient of thermal expansion) and mechanical properties (e.g., strength, hardness) between aluminum matrix composites and other metals. These disparities can give rise to defects such as thermal stress, distortion, and cracking during welding. Furthermore, brittle intermetallic compounds or weak bonding zones readily form at the weld interface, compromising both the strength and toughness of the joint, thereby degrading its overall quality and performance. In this regard, scholars both domestically and internationally have conducted extensive research. Aval et al. [11], explored the optimal process parameter window for friction stir welding of an A390-10 wt.% SiC composite and AA2024-T6 aluminum alloy. Sound weld quality was achieved at rotational speeds of 800 and 1000 rpm. However, at speeds below 800 rpm, surface grooves were observed in the weld due to insufficient material flow. Conversely, at speeds exceeding 1000 rpm, tunnel defects formed on the advancing side (AS) as a result of material turbulence. Tenali et al. investigated the effects of ultrasonic-assisted stir casting on the mechanical and tribological properties of particle-reinforced Al–Cu–Mg matrix composites. With the addition of 6 wt.% Palm Sprout ShellAsh (PSSA) (SiO_2_ and Al_2_O_3_), the AMC exhibited significant enhancements in tensile strength, compressive strength, and impact strength while suffering a substantial reduction in ductility [12]. Additionally, some researchers have attempted to improve joint quality by offsetting the tool towards the softer material side [13,14]. However, this approach proved to be largely ineffective. It has also been reported that placing the harder material on the advancing side (AS) enhances joint performance. For instance, Wert observed enhanced material mixing when positioning a 20 vol.% Al_2_O_3_/2014 aluminum composite on the AS [15].

To overcome the inadequate plasticity of aluminum matrix composites, this study proposes a bimetallic strategy employing a 15 vol.% SiC_p_/Al–Fe–V–Si composite as the wear-resistant layer and ZL101 aluminum alloy as the substrate layer. Leveraging the exceptional properties of SiC particulates—including high strength, elevated elastic modulus, and reduced coefficient of thermal expansion (CTE)—as reinforcing constituents. The joints of the above two materials will be processed via friction stir lap welding (FSLW). This design ensures wear resistance through SiC particles in the surface layer while leveraging the high toughness of the substrate to resist bulk fracture, thereby transcending the inherent “high-wear-resistance-implies-low-toughness” trade-off in conventional monolithic aluminum matrix composites. This work provided theoretical guidance for fabricating bimetallic brake disks with balanced wear resistance and toughness.

## 2. Materials and Methods

The 15 vol.% SiC_p_/Al–Fe–V–Si aluminum matrix composite used in this study was fabricated via powder metallurgy followed by hot extrusion processing. The detailed fabrication procedure was as follows: First, the 15 wt.% of SiC powder (with an average particle size of 20 μm) was uniformly mixed with a commercial Al–Fe–V–Si powder. The well-mixed powder was formed into a green body by cold isostatic pressing and then placed in a vacuum hot-press machine for sintering at 580 °C under 75 MPa for 4 h. The composite ingot was subsequently hot-extruded into a plate. The chemical composition of the Al–Fe–V–Si alloy is shown in Table 1. The other material used in the friction stir lap welding experiment is commercial ZL101 aluminum alloy, which is an aluminum alloy balancing castability, strength, corrosion resistance, and lightweight properties. The specific composition is shown in Table 1.

**Figure 1:**
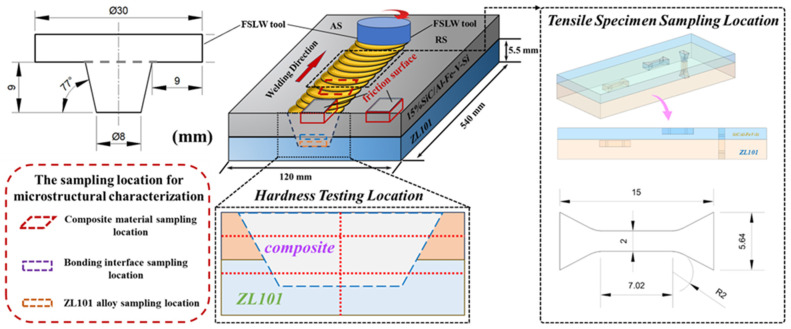
Schematic diagram of sampling for FSLW process and mechanical property testing.

The 15%SiC_p_/Al–Fe–V–Si aluminum matrix composite and ZL101 aluminum alloy were cut into plates with dimensions of 540 × 120 × 5.5 mm by wire electrical discharge machining. Before friction stir welding, the surfaces of the plates were ground and cleaned to ensure the bonding surfaces were flat, thus guaranteeing qualified welding quality. The 15%SiC_p_/Al–Fe–V–Si aluminum matrix composite plate was used as the upper sheet in lap welding, and the ZL101 aluminum alloy plate served as the lower sheet. After adjusting their positions, the sheets were fixed, and friction stir lap welding was carried out using a friction stir welding machine that was manufactured by CRRC Corporation Limited. The welding process is shown in Figure 1. The stirring tool used was H13 tool steel, and the tool rotated clockwise during welding. The specific experimental scheme is shown in Table 2. For convenience, the samples of the 15%SiC_p_/Al–Fe–V–Si aluminum matrix composite before and after friction stir lap welding (FSLW) were denoted as C-built, C-400, C-600, and C-800, corresponding to the as-extruded state and the samples processed at 400 rpm, 600 rpm, and 800 rpm, respectively. Similarly, the samples of the ZL101 aluminum alloy before and after FSLW were denoted as Z-built, Z-400, Z-600, and Z-800, corresponding to the original state and the samples processed at 400 rpm, 600 rpm, and 800 rpm, respectively.

To investigate the effect of the FSLW process on the mechanical properties of the samples, microhardness tests (Digital Display Vickers Microhardness Tester Model: HVS-1000, Shanghai Lidun Instrument & Meter Testing Technology Co., Ltd., Shanghai, China), tensile property tests (Instron-3382 Electronic Universal Testing Machine, Instron Corporation, Norwood, MA, USA), and friction and wear tests (CFT-I Comprehensive Surface Property Tester for Materials, Zhongke Kaihua Technology Development Co., Ltd., Lanzhou, China) were conducted. The microhardness tests were performed at the composite layer, ZL101 layer, and lap interface region of the joint. The distance between adjacent test points was 0.5 mm, with a test load of 2 N and a holding time of 15 s. The tensile tests were also conducted at the composite layer, ZL101 layer, and lap interface region of the joint, with a tensile rate of 0.5 mm/min. A ball-on-disk tribological test was conducted to investigate the friction and wear properties of the 15%SiC_p_/Al–Fe–V–Si aluminum matrix composite. The friction radius was fixed at 3 mm; the total friction distance was set to 200 m; the load was maintained at 12 N; and the rotational speed was set at 200 rpm. Specific experimental parameters are listed in Table 3. The sampling locations and dimensions are shown in Figure 1.

The microstructures of the 15%SiC_p_/Al–Fe–V–Si aluminum matrix composite and ZL101 aluminum alloy under different conditions were observed by a Optical Metallographic Microscope (CK-300 Optical Metallographic Microscope, Shanghai Caikang Optical Instruments Co., Ltd., Shanghai, China). Meanwhile, a FEI QUANTA 200 Scanning Electron Microscope (FEI Company, Hillsboro, OR, USA) was used to observe the 15%SiCp/Al–Fe–V–Si aluminum matrix composite and ZL101 aluminum alloy in different states, including microstructures, friction and wear morphologies, and tensile fracture surfaces. Additionally, a transmission electron microscope (TEM-FEI Tecnai G2 F20, FEI Company, Hillsboro, OR, USA) and an X-ray diffractometer (XtalLAB PRO, Rigaku, Japan) were employed to characterize the phase composition and transformations of the materials. The TEM samples were prepared by ion thinning. The specific sampling locations are shown in Figure 1.

## 3. Results and Discussion

### 3.1. Microstructural Observation of FSLW Joints

#### 3.1.1. Phase Analysis

**Figure 2:**
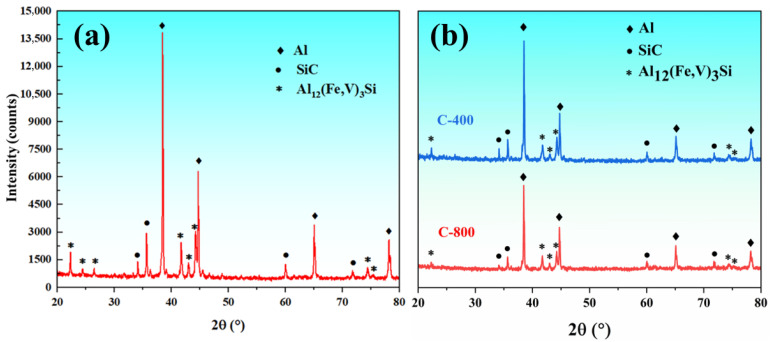
XRD pattern of 15%SiC_p_/Al–Fe–V–Si composite material: (**a**) extruded state; (**b**) FSLW state.

**Figure 3:**
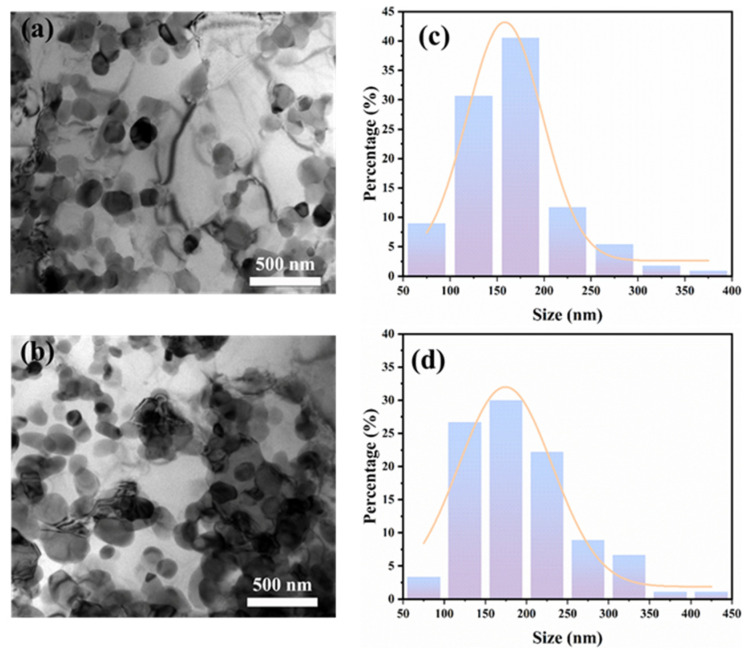
TEM characterization of 15%SiC_p_/Al–Fe–V–Si composite before and after FSLW: (**a**) C-built; (**b**) C-800; (**c**) size distribution of Al_12_(Fe,V)_3_Si in C-built; (**d**) size distribution of Al_12_(Fe,V)_3_Si in C-800.

Figure 2 shows the XRD patterns of as-extruded 15%SiC_p_/Al–Fe–V–Si aluminum matrix composites and the aluminum matrix composite layer after friction stir lap welding (FSLW). It can be seen that the as-extruded composites consist of Al, Al_12_(Fe,V)_3_Si, and SiC phases. After FSLW, the diffraction peaks of the Al_12_(Fe,V)_3_Si heat-resistant phase still exist in the 15%SiC_p_/Al–Fe–V–Si composite at rotational speeds of 400 rpm and 800 rpm [16]. This indicates that the high temperature and severe plastic deformation during FSLW did not force the decomposition of the heat-resistant phase. Additionally, as shown in Figure 3a,b the Al_12_(Fe,V)_3_Si phase did not significantly coarsen after FSLW. The particle size distribution reveals that the size of the Al_12_(Fe,V)_3_Si phase is mostly around 150 nm, suggesting that the short thermal cycle during FSLW is insufficient to drive the coarsening of the heat-resistant phase.

#### 3.1.2. Macro Morphology of Weld Seam

**Figure 4:**
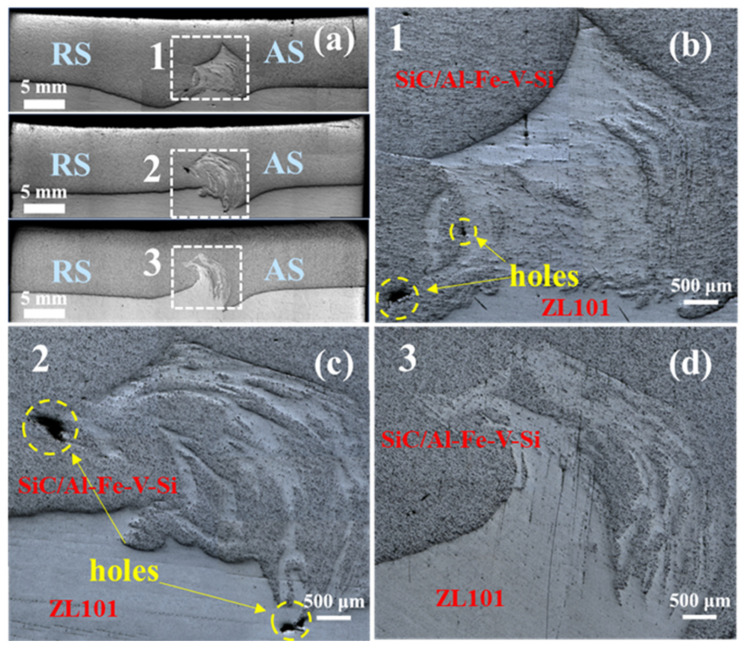
Microstructures of weld cross-sections for samples at different FSLW rotational speeds: (**a**) macro morphology; (**b**) C-400; (**c**) C-600; (**d**) C-800.

Figure 4a shows the macro morphology of the joint cross-sections of samples prepared at different FSLW rotational speeds. Unlike most literature reports [17,18,19], clear boundaries were not formed between the base metal, heat-affected zone, thermo-mechanically affected zone, and weld zone in the joint cross-sections. The absence of obvious regional boundaries may indicate a small mechanical property gradient in the joint, reducing the risk of softening or embrittlement in the heat-affected zone, which needs to be further verified by tensile and hardness tests. It can also be observed that the lower ZL101 aluminum alloy was stirred into the aluminum matrix composite layer under the action of the stirring tool. This phenomenon may be caused by the excessively long stirring pin on the one hand, and by the physical property differences between the two metals on the other hand. In addition, the material flow distribution between the advancing side and the retreating side was significantly different, that is, the position where ZL101 aluminum alloy was stirred in was all located on the advancing side.

Further magnification observation of the weld seam (Figure 4b,c) revealed that due to the low FSLW rotational speed, the metal fluidity was poor, leading to void defects of varying sizes at the weld bottom. When the rotational speed increased to 800 rpm, the heat input improved the metal fluidity significantly, and no obvious porosity was observed in Figure 4d, indicating that a higher rotational speed helps the metal fill the cavity region in a timely manner, thus improving the quality of the welded joint. It is worth noting that void defects mostly appeared near the lap interface and were predominantly present in the 15%SiC_p_/Al–Fe–V–Si composite layer. This phenomenon suggests that the 15%SiC_p_/Al–Fe–V–Si composite, due to the strengthening effect of heat-resistant phases and reinforcing particles, has higher heat resistance than ZL101 aluminum alloy, resulting in weaker fluidity than ZL101, which facilitates the formation of void defects at the interface.

#### 3.1.3. Microstructure of Weld Seam

**Figure 5:**
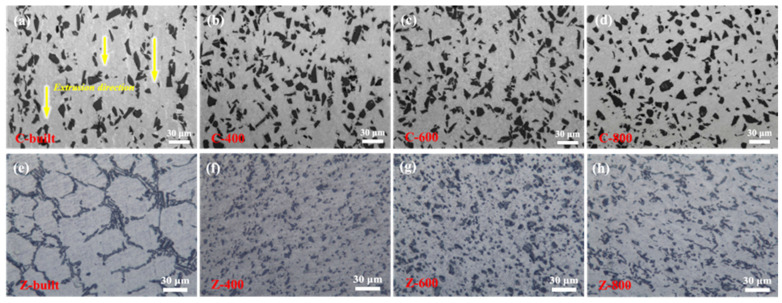
Metallographic microstructure comparison of materials before and after FSLW: (**a**–**d**) 15%SiC_p_/Al–Fe–V–Si composite; (**e**–**h**) ZL101 aluminum alloy.

Figure 5 shows the metallographic images of the materials before and after FSLW. It can be observed that the SiC particles in the C-built specimen are distributed along the extrusion direction in a streamline shape, leading to the formation of large “SiC-poor zones” between two SiC streamlines. The inhomogeneity of the microstructure may cause deterioration of certain properties. However, after FSLW, the SiC particles in the 15%SiC_p_/Al–Fe–V–Si composite tend to distribute uniformly, and the number of fine-sized particles increases, which significantly improves the microstructural homogeneity. During severe plastic deformation, SiC particles can only coordinate the deformation of the material by rotating or moving, and this inhomogeneous deformation behavior causes the redistribution of reinforcing particles in the matrix. In the FSLW process, the stirring action of the stirring tool promotes the crushing and redistribution of SiC particles [20,21].

**Figure 6:**
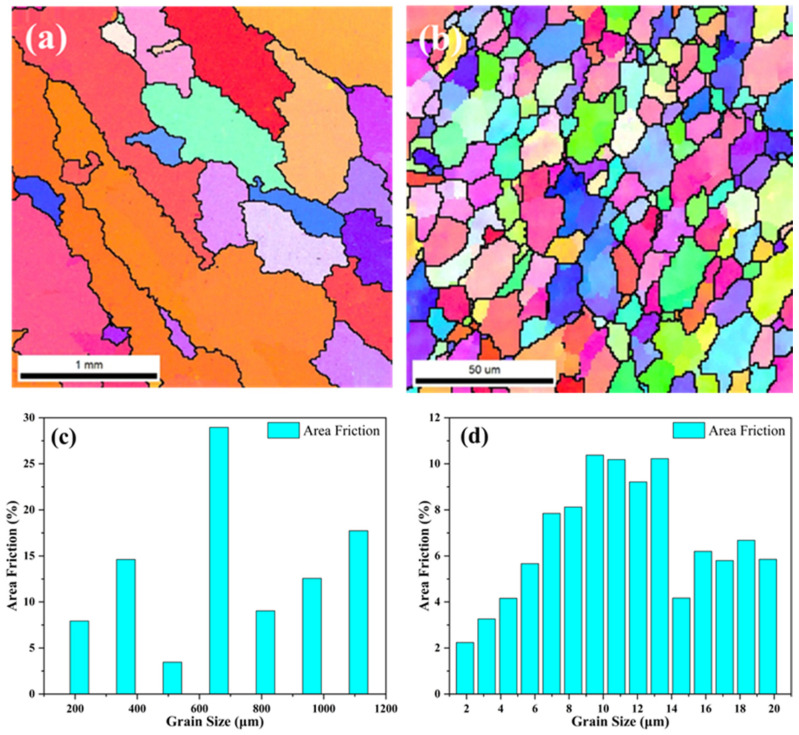
EBSD microstructure comparison of ZL101 aluminum alloy layer before and after friction stir welding: (**a**) Z-built; (**b**) Z-800; (**c**) particle size histogram of Z-build; (**d**) particle size histogram of Z-800.

For the Z-built specimen, the eutectic phases exhibit coarse lamellar or acicular shapes and distribute along the grain boundaries, significantly affecting the mechanical properties of the alloy. Since eutectic phases (such as eutectic silicon in Al–Si alloys or θ phase in Al–Cu alloys) are typically hard and brittle, their continuous or network distribution at grain boundaries forms local stress concentration points, which easily become preferential paths for crack initiation and propagation under external forces, leading to a decrease in the ductility and toughness of the material. Meanwhile, the grain boundary eutectic phases can hinder dislocation slip, slightly increasing the strength but exacerbating the tendency for brittle fracture. However, the microstructure of ZL101 aluminum alloy after FSLW has undergone significant changes. FSLW promotes the morphological transformation of eutectic phases through local heating and plastic deformation. Due to the action of the stirring tool, a large number of eutectic phases are fragmented and dispersed uniformly in the matrix. The eutectic phases transform from a continuous network distribution to fine granular state, reducing stress concentration and improving microstructural homogeneity, which helps to suppress crack initiation and simultaneously enhances ductility and toughness. On the other hand, the fragmented eutectic phases embedded within or at grain boundaries contribute to strengthening the grain boundary pinning effect, thereby inhibiting abnormal grain growth. Figure 6 shows the EBSD images of the ZL101 aluminum alloy layer before and after friction stir welding. It can be observed that the Z-built specimen has coarse grains, which affect the mechanical properties of the alloy. However, after friction stir welding, the grains of ZL101 alloy are significantly refined, indicating that during FSLW, the ZL101 aluminum alloy undergoes severe plastic deformation under the combined action of continuous shearing and frictional heat, storing more strain energy and undergoing dynamic recrystallization, thus refining the grains. Grain refinement increases the number of grain boundaries, which helps to hinder crack propagation and enhance fatigue resistance while also endowing better plasticity, beneficial for further improving the plasticity of the bimetallic composite.

**Figure 7:**
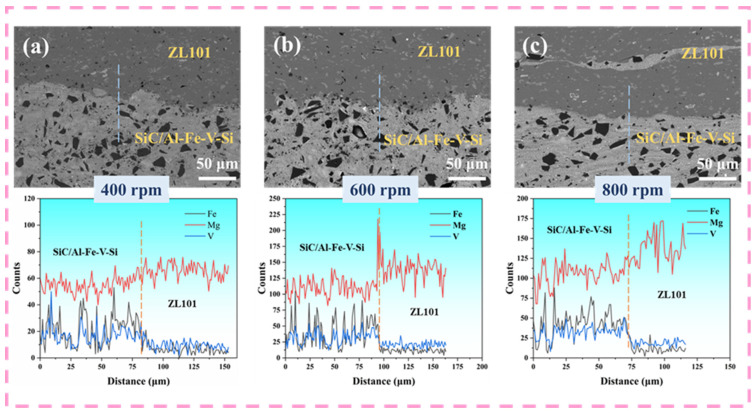
SEM images and elemental line scans of the weld-lap interface at different FSLW rotational speeds: (**a**) 400 rpm; (**b**) 600 rpm; (**c**) 800 rpm.

Figure 7 shows the SEM microstructures and EDS line scanning analysis results of the bimetallic lap interface after FSLW processing at different rotational speeds. Microstructural analysis indicates that the joint interfaces under the three process parameters exhibit good metallurgical bonding characteristics, with no macroscopic defects such as voids or microcracks observed. Elemental distribution characteristics show that the Mg element presents a gradient transition trend at the interface between the 15%SiC_p_/Al–Fe–V–Si composite layer and the ZL101 aluminum alloy layer, confirming the occurrence of elemental interdiffusion in the interface region and indicating the potential formation of metallurgical bonding between heterogeneous materials. In contrast, the concentration distributions of Fe and V elements exhibit a stepwise abrupt change at the interface, a special pattern originating from the solid solution of Fe and V primarily in the Al_12_(Fe,V)_3_Si heat-resistant intermetallic compound phase. According to the research by He et al. [22], this heat-resistant phase maintains excellent structural stability without significant coarsening after isothermal aging at 550 °C for 200 h, confirming its superior thermal stability. This property directly leads to the difficulty of Fe and V elements dissociating and diffusing from the Al_12_(Fe,V)_3_Si phase during the thermo-mechanical coupling process of FSLW.

### 3.2. Mechanical Properties of FSLW Joints

#### 3.2.1. Microhardness

**Figure 8:**
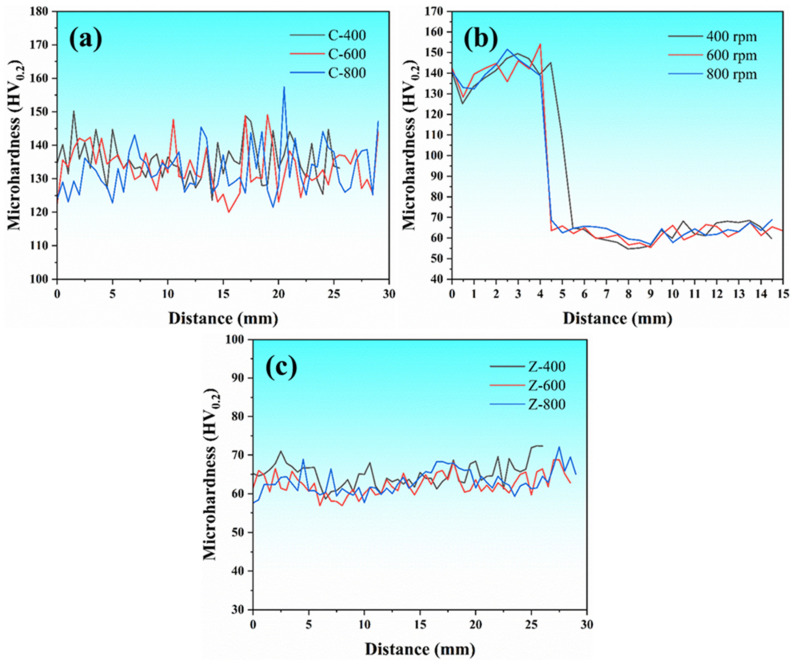
Comparison of microhardness distributions for samples at different FSLW rotational speeds: (**a**) 15%SiC_p_/Al–Fe–V–Si composite; (**b**) lap interface; (**c**) ZL101 aluminum alloy.

Figure 8 shows the microhardness distribution characteristics of various zones in FSLW joints under different welding parameters. Figure 8a corresponds to the hardness distribution of the 15%SiC_p_/Al–Fe–V–Si composite layer. Experimental data show that the rotational speed of the stirring tool has no significant effect on the material hardness. The hardness curves under the three parameters of C-400, C-600, and C-800 all fluctuate horizontally, with the range around 135 HV, which is equivalent to the hardness of the base metal. This phenomenon indicates that a significant heat-affected zone (HAZ) was not formed during FSLW, mainly due to the excellent high-temperature resistance of the 15%SiC_p_/Al–Fe–V–Si composite, where the short thermal cycle during welding is insufficient to soften the metal. It should be specifically noted that the discrete characteristics of the hardness values in the composite layer mainly stem from the size effect of Vickers indentation testing—local strengthening response occurs when the indenter contacts the boundary region of SiC reinforcing particles [23,24,25]. Figure 8c shows the hardness evolution law of the ZL101 aluminum alloy layer, where the hardness values under different rotational speeds all stabilize in the range of 60–70 HV, confirming that the thermo-mechanical loading of FSLW does not cause significant hardening or softening of the aluminum alloy matrix. It is worth noting that the hardness distribution curve perpendicular to the lap interface in Figure 8b shows an obvious abrupt hardness gradient at the interface between the composite layer (135 HV) and the aluminum alloy layer (65 HV), which directly reflects the significant difference in mechanical properties between heterogeneous materials.

#### 3.2.2. Tensile Property

**Figure 9:**
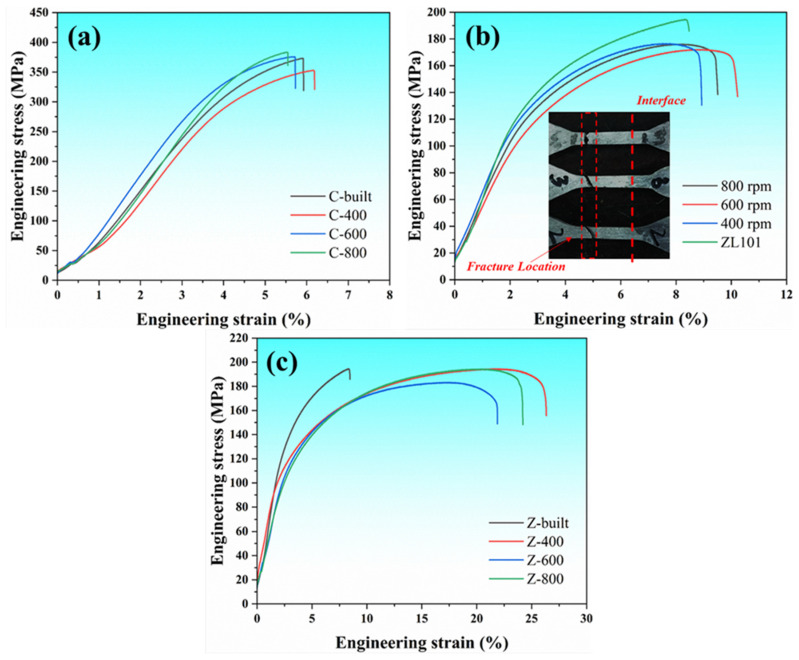
Engineering stress-engineering strain curves of materials before and after FSLW: (**a**) 15%SiC_p_/Al–Fe–V–Si composite; (**b**) lap interface; (**c**) ZL101 aluminum alloy.

Figure 9a compares the engineering stress–engineering strain curves of the 15%SiC_p_/Al–Fe–V–Si composite layer before and after FSLW. It can be seen that no obvious difference is exhibited between them. On the one hand, although the severe plastic flow during FSLW may cause fragmentation or redistribution of some SiC particles, the overall volume fraction does not significantly decrease, so the secondary phase strengthening effect is retained, supporting the tensile strength to stabilize above 350 MPa. On the other hand, during the FSLW process, although frictional heating raises the local temperature to 200–600 °C, the heat-resistant phases do not undergo obvious coarsening or decomposition, thereby maintaining the matrix’s softening resistance [26]. This indicates that within a certain parameter range, the friction stir lap welding process has little effect on the tensile strength of the 15%SiC_p_/Al–Fe–V–Si composite. It also shows that the 15%SiC_p_/Al–Fe–V–Si composite has a certain degree of thermal stability, and its performance will not deteriorate due to frictional heating during FSLW. This characteristic makes it particularly suitable for high-temperature components that require a balance of lightweight, high strength, and welding reliability.

Figure 9b shows the engineering stress-engineering strain curves of the friction stir lap welding (FSLW) interface, revealing the mechanical response characteristics and fracture behavior of the bimetallic joint. Key phenomena and mechanisms observed from the curves are as follows: All specimens fractured in the ZL101 aluminum alloy region rather than at the interface or on the composite side, indicating that the lap interface formed sufficient metallurgical bonding and mechanical interlocking during welding. The interface bonding strength exceeds the bearing capacity of the ZL101 aluminum alloy matrix, causing fracture to preferentially occur in the relatively weaker aluminum alloy region. Since the strength of the aluminum matrix composite (15%SiC_p_/Al–Fe–V–Si) is significantly higher than that of ZL101 aluminum alloy, the applied load tends to concentrate stress on the low-strength aluminum alloy side, accelerating its plastic instability and fracture.

Figure 9c presents the engineering stress–engineering strain curves of the ZL101 layer before and after FSLW. The tensile strength shows no significant difference before and after lap welding, but it is noteworthy that the elongation of the welded ZL101 alloy is significantly improved. As previously discussed, Z-built samples have coarse grains and a large number of brittle acicular eutectic phases around the grains, which may cause the eutectic phases to dissever the matrix and promote crack propagation during tension, leading to reduced elongation [27,28,29]. In contrast, after FSLW, the eutectic phases in ZL101 aluminum alloy are refined into fine particles and uniformly dispersed in the matrix, which helps reduce stress concentration and inhibit crack initiation. Moreover, the refined grains after FSLW slow down the crack propagation rate, resulting in a significant increase in elongation (≥20%). Relative to the ultrasonic-assisted stir casting (UASC) process developed by Tenali et al.—which substantially enhances the strength of aluminum matrix composites (AMCs) at the expense of significant ductility degradation—the strategic integration of UASC with friction stir processing (FSP) demonstrates potential for synergistic performance optimization. This hybrid approach is projected to deliver dramatic strength improvements while preserving ductility, thereby overcoming the characteristic strength–ductility trade-off in particulate-reinforced AMCs.

### 3.3. Friction and Wear Behavior of the Composites

**Figure 10:**
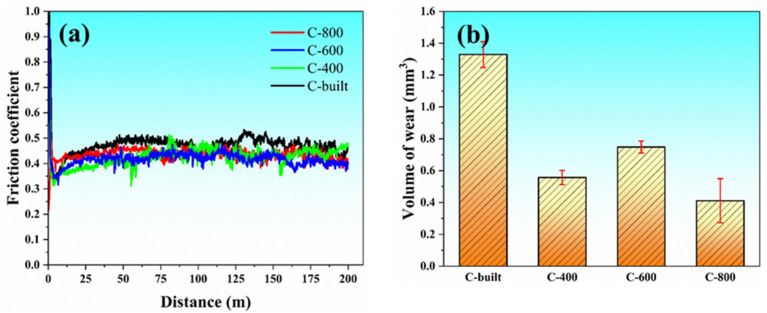
Friction coefficient and wear amount of the 15%SiC_p_/Al–Fe–V–Si composite before and after FSLW: (**a**) friction coefficient; (**b**) wear amount.

The friction coefficient is an important parameter for measuring the friction and wear characteristics of materials and exploring friction and wear mechanisms. Its value depends not only on the dynamic changes in the friction process but also closely relates to the material properties and surface conditions of the friction pair. Figure 10 shows the friction coefficient curves and wear amount statistics of as-extruded samples and 15%SiC_p_/Al–Fe–V–Si composites prepared by three different FSLW process parameters. As seen in Figure 10a, the friction coefficient of the composite exhibits significant fluctuations in the initial wear stage, indicating that the material is in the running-in phase; as the friction stroke increases, the friction coefficient gradually stabilizes and enters the steady-state wear stage. It is worth noting that the friction coefficient of the as-extruded sample remains at a high level (approximately 0.5), while that of the samples processed by FSLW is significantly reduced (average approximately 0.45), and there is little difference in the friction coefficients among the three process parameter samples (C-400, C-600, C-800).

In addition, as shown in Figure 10b, the wear amount of the as-extruded sample is significantly higher than that of the friction-stirred samples, reaching 1.3 mm^3^, confirming that the friction stir welding process can effectively improve the friction and wear resistance of the 15%SiC_p_/Al–Fe–V–Si composite. It is noteworthy that the wear amounts of samples prepared at three different rotational speeds (400, 600, 800 rpm) do not show obvious statistical differences, indicating that within this parameter range, the FSLW rotational speed has no significant effect on the friction and wear properties of the composite.

**Figure 11:**
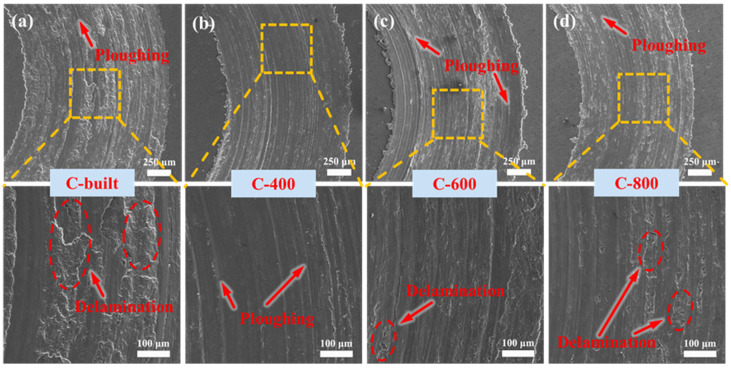
Worn surface morphologies of the 15%SiC_p_/Al–Fe–V–Si composite under room-temperature friction and wear before and after FSLW: (**a**) C-built; (**b**) C-400; (**c**) C-600; (**d**) C-800.

To reveal the differences in friction and wear mechanisms among samples in different states, the worn surface morphologies of each specimen were characterized by SEM (Figure 11). Microscopic analysis shows that the wear tracks of the as-extruded sample (Figure 11a) exhibit typical plowing morphology and large-area lamellar delamination. High-magnification imaging reveals that the delamination pits are deep, indicating the coexistence of abrasive wear and severe delamination wear during the wear process. Figure 11b–d show the wear morphologies of samples processed by three friction stir welding processes, all of which exhibit similar wear mechanisms: the worn surfaces are dominated by plowing features with slight delamination. Observed at high magnification, these delaminations are small in area and shallow in depth. It can be inferred that the main wear mechanism of the friction stir welded samples is abrasive wear accompanied by mild delamination wear. The worn surface features of the four types of samples are consistent with the wear amount results, confirming that large-area delamination wear is the primary cause of severe wear in the as-extruded sample.

**Figure 12:**
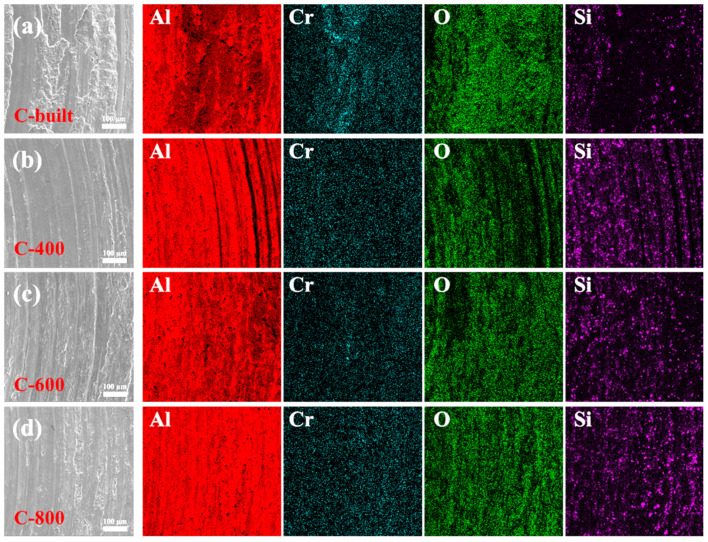
EDS surface scanning results of worn tracks for the 15%SiC_p_/Al–Fe–V–Si composite under room-temperature friction and wear before and after FSLW: (**a**) C-built; (**b**) C-400; (**c**) C-600; (**d**) C-800.

To further explore the wear mechanisms of the four types of samples, EDS surface scanning analysis was performed on the worn surfaces. Figure 12 shows that all samples exhibit obvious oxygen element enrichment on the worn surfaces, indicating the occurrence of oxidative wear during room-temperature wear. The formation of oxidation products is beneficial for forming a barrier layer at the friction pair interface, effectively reducing direct contact between the grinding ball and the sample surface to achieve anti-friction effects [30,31,32]. It is also noteworthy that the SiC particles in the as-extruded sample are sparsely distributed, while those on the worn surfaces of FSLW-treated samples are relatively uniform and dense. As previously analyzed, the SiC particles in the as-extruded specimen exhibit a streamline distribution along the extrusion direction (Figure 5a), leading to the formation of wider “SiC-poor zones” between adjacent particle streamlines. During frictional contact, the grinding ball preferentially wears the Al matrix in the “SiC-poor zones”, causing surface SiC particles to rapidly detach due to lack of matrix anchoring, which reduces the number of SiC particles on the worn surface. On the other hand, the “Si-poor zones” undergo plastic deformation under contact stress. Since the Al–Fe–V–Si matrix itself has low ductility and toughness, micro-cracks initiated during deformation propagate along the SiC streamline direction, eventually forming flake-like delaminations. Based on the above two points, the as-extruded sample finally suffers from large-area delamination on the worn surface during friction and wear. The loss of SiC support also results in a higher friction coefficient. In sharp contrast, the FSLW specimens undergo intense plastic flow from the stirring tool, successfully achieving secondary uniform distribution of SiC particles (Figure 5b–d), effectively eliminating the “SiC-poor zones”. This uniform distribution of reinforcing phases forms a continuous network-like SiC support structure on the worn surface, reducing the contact area between the grinding ball and the matrix, which is ultimately beneficial for enhancing wear resistance.

**Figure 13:**
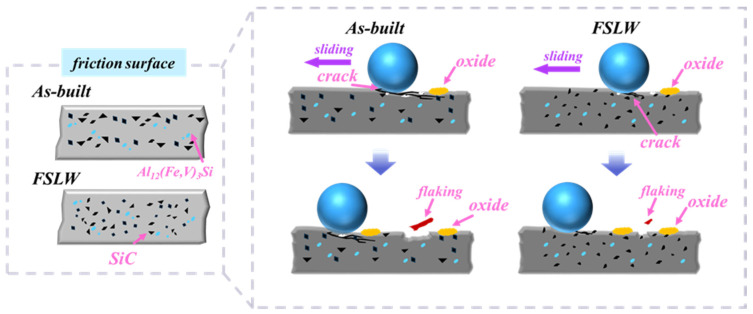
Schematic diagram of friction and wear mechanisms.

Comprehensive analysis shows that the room-temperature friction and wear mechanisms of specimens before and after friction stir lap welding (FSLW) exhibit significant differences (Figure 13). For the as-extruded sample, due to the presence of “SiC-poor zones”, these regions directly contact the grinding ball during friction and wear, prone to crack propagation and grinding off under repeated deformation, thereby exposing large areas of SiC particles to be pulled out. The pulled-out hard particles then continue to wear the matrix. The loss of SiC particle support causes large-scale material delamination, and the extensive delamination is the main cause of the high wear amount in the as-extruded sample. However, after FSLW, the distribution of SiC particles tends to be uniform with an increase in fine particles, which greatly improves microstructural uniformity and reduces stress concentration. Thus, it can provide effective support during friction, enhancing wear resistance. The worn tracks are dominated by shallow plowing features with minor shallow delamination. In addition, when friction and wear occur, all samples are accompanied by oxidative wear due to frictional heating [33].

### 3.4. Future Research Directions

This study addresses the critical challenge of strength–toughness enhancement in aluminum matrix composite (AMC) brake disks. We conducted a systematic investigation into the friction stir lap welding (FSLW) process for joining the 15 vol.% SiCp/Al–Fe–V–Si composite and the ZL101 cast aluminum alloy. A preliminary processing window compatible with both materials was established. However, significant limitations remain in the current research. To advance the development and practical application of FSW for AMC brake disks, future work will focus on the following critical research avenues:

(1) Process Parameter Optimization and Multi-Factor Coupling Analysis
  Parameter Scope Expansion: Beyond rotational speed, a comprehensive investigation into the effects of welding speed, axial force (plunge depth), tool tilt angle, and dwell time on weld morphology, interfacial bond strength, and fracture toughness is imperative. This necessitates establishing robust process–property relationships through quantitative modeling. Synergistic Parameter Interactions: The coupled effects of rotational speed with other key parameters require rigorous analysis. Orthogonal experimental design or Response Surface Methodology (RSM) will be employed to elucidate these interactions and optimize the robust process window.
(2) Multi-Pass FSLW for Scalability
  Current findings are limited to single-pass lap welds. To meet the dimensional requirements of practical brake disks, research must extend to multi-pass FSLW strategies. The primary focus will be characterizing the influence of interpass thermal history on microstructural evolution and resultant mechanical behavior. This understanding is crucial for providing the theoretical foundation and technical readiness required for lightweight, high-performance braking systems.
(3) Comprehensive Performance Evaluation Framework
  Tribological Assessment: Moving beyond simplistic pin-on-disk tests, performance evaluation must utilize disk-on-disk tribo-pairs under realistic conditions, incorporating variations in temperature, humidity, and braking pressure. This will elucidate the evolution of friction coefficients, wear rates, and dominant wear mechanisms.
  Environmental Durability: The long-term stability and corrosion resistance of welded joints must be assessed under aggressive environments (e.g., salt spray, high humidity/temperature). Dynamic Mechanical Integrity: Evaluation under service-relevant dynamic loading is essential. This includes assessing fracture toughness under high-speed impact and fatigue life under cyclic loading conditions to ensure structural reliability.

## 4. Conclusions

This study efficiently achieved the joining of the 15%SiC_p_/Al–Fe–V–Si composite and ZL101 aluminum alloy by friction stir lap welding (FSLW), and then studied the microstructure and mechanical properties of the joints and the friction and wear mechanisms of the FSLW composites.

(1) Insufficient heat input at low rotational speeds (400–600 rpm) led to uneven material mixing and internal void defects in the joint, while high rotational speed (800 rpm) enhanced plastic material flowability, significantly improving interface bonding quality with little defects.
(2) FSLW promoted uniform redistribution of SiC particles, improving microstructural homogeneity. The tensile strength was comparable to the base metal (maintaining over 350 MPa) and hardness remained 135 HV, indicating that the FSLW process did not deteriorate the mechanical properties of the 15%SiC_p_/Al–Fe–V–Si composites. The ZL101 aluminum alloy underwent grain refinement via recrystallization, with eutectic silicon crushed and dispersed, increasing elongation to 22%. The interface bonding strength exceeded that of the aluminum alloy matrix, with tensile fracture occurring exclusively in the ZL101 side, achieving 85% of the ZL101 base metal’s tensile strength. Comprehensive analysis of microstructure and mechanical properties showed that samples prepared at 800 rpm rotational speed and 100 mm/min travel speed exhibited optimal overall performance.
(3) FSLW treatment reduced the wear amount of the composite from 1.3 mm^3^ (as-extruded) to 0.8 mm^3^, transforming the wear mechanism from delamination wear (as-extruded) to dominant abrasive wear. The uniformly distributed SiC particles formed an effective anti-wear support network, leading to a decrease in friction coefficient and improving wear resistance.

## Figures and Tables

**Table 1:** Chemical composition of the alloy (wt.%).

Alloy	Si	Fe	V	Mg	Mn	Zn	Al
Al–Fe–V–Si alloy	1.95	7.75	1.04	0.013	0.031	-	balance
ZL101 aluminum alloy	6.7	0.107	-	0.446	0.0012	0.021	balance

**Table 2:** Parameter settings for friction stir lap welding.

Code	Travel Speed (mm/min)	Rotational Speed (rpm)	Plunge Depth (mm)	Tilt Angle (°)
400 rpm	100	400	0.3	2.5
600 rpm	100	600	0.3	2.5
800 rpm	100	800	0.3	2.5

**Table 3:** Friction and wear experimental scheme.

Sample	Friction and Wear Parameters
C-built	Load: 12 N Rotational Speed: 200 rpm Counter-Body: 304 Stainless Steel Ball
C-400	
C-600	
C-800	

## Data Availability

The original contributions presented in this study are included in the article. Further inquiries can be directed to the corresponding authors.
